# Immune reconstitution disease associated with parasitic infections following antiretroviral treatment

**DOI:** 10.1111/j.1365-3024.2006.00900.x

**Published:** 2006-11

**Authors:** S D LAWN, R J WILKINSON

**Affiliations:** 1 The Desmond Tutu HIV Centre, Institute for Infectious Disease and Molecular Medicine, Faculty of Health Sciences, University of Cape Town Cape Town, South Africa; 2 Clinical Research Unit, Department of Infectious and Tropical Diseases, London School of Hygiene and Tropical Medicine London, UK; 3 Mycobacterial Immunology Group, Institute for Infectious Disease and Molecular Medicine, Faculty of Health Sciences, University of Cape Town Cape Town, South Africa; 4 Wellcome Trust Centre for Research in Clinical Tropical Medicine, Division of Medicine, Imperial College London London, UK

**Keywords:** antiretroviral treatment, HAART, HIV, immune reconstitution inflammatory syndrome, immune reconstitution, immune restoration, IRIS, parasitic infection, tropical infection

## Abstract

HIV-associated immune reconstitution disease (IRD) is the clinical presentation or deterioration of opportunistic infections that results from enhancement of pathogen-specific immune responses among patients responding to antiretroviral treatment (ART). The vast majority of reported cases of IRD have been associated with mycobacterial, chronic viral and invasive fungal infections; such cases result from dysregulated augmentation of cell-mediated type 1 cytokine-secreting host immune responses. However, the spectrum of infections now recognized as associated with IRD is expanding and includes a number of parasitic infections, which may be mediated by different immunopathological mechanisms. These include leishmaniasis (visceral, cutaneous, mucosal and post kala azar dermal leishmaniasis), schistosomiasis and strongyloidiasis. Since the major burden of HIV lies in resource-limited countries where access to ART is now rapidly expanding, increased awareness and knowledge of these phenomena is important. Here we review the clinical spectrum and pathogenesis of IRD associated with parasitic infections.

## INTRODUCTION

HIV-associated morbidity and mortality has dramatically decreased in many high-income countries since the advent of antiretroviral treatment (ART) in the mid 1990s (1–3). Robust suppression of viral replication by ART permits both quantitative and functional reconstitution of the immune system (4–6). As a result, primary and secondary prophylaxis for many opportunistic infections that occur among those with advanced immunodeficiency can often be discontinued (7). However, the initial rapid phase of immune recovery may also directly result in adverse clinical phenomena. Previously subclinical infections may be ‘unmasked’ or pre-existing partially treated opportunistic infections may clinically deteriorate (8–10). Such phenomena are believed to arise from a dysregulated augmentation of the host inflammatory response to these infections. Various terms have been used to refer to this, including ‘immune reconstitution syndrome’, ‘immune restoration disease’, and the ‘immune reconstitution inflammatory syndrome’ (familiarly abbreviated to ‘IRIS’). In this review we have used the term ‘immune reconstitution disease’ (IRD).

IRD is not a new phenomenon, but has long been recognized as a complication occurring among patients with severe immunosuppression in whom immune function is rapidly restored. Thus, for example, IRD may occur following withdrawal or rapid dosage reduction of corticosteroid treatment and among patients in whom the blood neutrophil count recovers following cytotoxic chemotherapy or bone marrow transplantation (11). The pathogens involved in IRD reflect the spectrum of opportunistic infections associated with the specific form of immunosuppression. For example, IRD following recovery from neutropenia is typically associated with fungal and pyogenic infections (11).

The vast majority of cases of IRD associated with ART have been reported from high-income countries and have been associated with a wide range of non-parasitic opportunistic infections, including: (a) bacteria (*Mycobacterium tuberculosis*, *Mycobacterium avium* complex and other non-tuberculous mycobacteria); (b) viruses (cytomegalovirus, varicella zoster virus, herpes simplex virus, human herpes virus-8, hepatitis B and C and JC virus); and (c) fungi (*Pneumocystis jirovecii*, *Cryptococcus neoformans and Histoplasma* spp.) (8–10). However, the spectrum of infections recognized to be associated with IRD continues to increase and case reports now describe IRD associated with some parasitic infections (*vide infra*).

The majority of people with HIV/AIDS live in resource-limited countries and in June 2005 WHO estimated that 6·5 million people living in such countries were in urgent need of ART (12). Despite formidable logistical challenges, access to ART is now expanding. However, patients in resource-limited settings typically enter ART programmes with advanced symptomatic disease and very low blood CD4 cell counts (13,14). This predisposes them to high rates of both clinical and subclinical opportunistic infections that may potentially be associated with IRD. To date, IRD associated with tuberculosis and cryptococcocal meningitis is reported to be associated with the greatest burden of morbidity and mortality in sub-Saharan Africa (13,15). However, the prevalence of chronic parasitic infections is very high in these populations and yet very little is known about whether these infections may either be associated with IRD or may perhaps modulate immune responses involved in IRD associated with other pathogens. In this paper we review what is currently known about the clinical manifestations and pathogenesis of IRD associated with parasitic infections.

## DEFINITION OF IRD

IRD can be defined as the clinical presentation or deterioration of opportunistic infections that results from enhancement of pathogen-specific immune responses among patients responding to ART. However, diagnosis in practice is not straightforward, being one of clinical judgement based on various lines of indirect evidence. These may include: (a) the clinical manifestation or pattern of progression of an opportunistic infection that is unusual; (b) a temporal relationship with ART initiation; (c) exclusion of alternative explanations; (d) demonstrated efficacy of ART (e.g. reduction in viral load or rise in CD4 cell count; (e) evidence of improved CD4 cell function (e.g. development of a positive tuberculin skin test); and (f) histopathology consistent with the diagnosis. Competing explanations for these clinical manifestations that should be excluded are the occurrence of opportunistic infections as a result of residual immunodeficiency and inadequate treatment of an opportunistic infection, including that resulting from drug resistance.

## IMMUNOLOGICAL EFFECTS OF ART

Successful ART is associated with a rapid (> 90%) reduction in plasma viral load within the first weeks of ART. The most characteristic immunological feature of HIV infection is depletion of CD4 T cell numbers, and restoration of the CD4 cell subset during ART appears to occur in two principal phases. The initial rapid phase of CD4 cell recovery can usually be detected within the first 1–2 weeks of starting treatment and extends over 2–3 months (4,5). Data suggest that this phase largely represents a redistribution of activated CD4^+^CD45RO^+^ memory cells previously sequestered in lymphoid tissue and a reduction in apoptotic cell death (4,5,16,17). Those with the greatest pretreatment viral loads and CD4 decline have the greatest rates of phase 1 CD4 cell count recovery (18–21). A slower second phase of CD4 cell expansion persists for 1–2 years with variable smaller increments occurring thereafter. This second thymus-dependent phase is associated with expansion of naïve CD45RA^+^CD62L^+^ cells (4,22).

The increase in circulating CD4 cell numbers is also associated with an improvement in effector function, the extent of which is directly related to the degree of viral load suppression and the CD4 cell counts in the longer term (6). IL-2-mediated T lymphocyte proliferative responses to recall antigens are restored (23,24). A switch from type 2 to type 1 cytokine profiles in T lymphocyte stimulation assays and in tissues is detectable early in treatment, with increases in IFN-γ and IL-2 production in response to antigen (25,26). There is diversification of the pathogen-specific T cell receptor repertoire (27,28) and delayed-type hypersensitivity responses to antigens assessed by skin testing are restored (24,29).

Although HIV is principally characterized by CD4 cell depletion, functional deficits in other cells of the innate and acquired immune systems also occur. However, the literature concerning effects of ART on other cell types is much less comprehensive than that concerning CD4 cell reconstitution. Moreover, many of these effects are CD4 cell-dependent, so that attributing ART-induced improvements in immune function to either CD4 cells or to other cell types is difficult.

Decreased macrophage function, including impaired chemotaxis, binding of microorganisms, phagocytosis, antigen processing, microbicidal activity and capacity to secrete interleukin-12 is recognized (30–34). However, data on the impact of ART on these functions are lacking. Dendritic cells (DC) in their role as antigen-presenting cells exert a substantial influence on the phenotype of the subsequent acquired immune response. Both plasmacytoid (pDC) and myeloid (mDC) subsets are reduced in numbers in HIV infection. During ART there is evidence that the defect in pDC is only partially reversible (35,36), whereas the mDC subset appears better reconstituted by ART (35–37). This differential recovery is of interest when considering IRD because the mDC subset is responsible for polarizing acquired response towards type 1 responses. Reduced CD4^+^ T cell counts are also associated with decreased circulating CD3^−^/CD16^+^/CD56^+^ NK cells (38), however, ART does not appear to be associated with an increase in NK cell function (38–41).

CD8 activation (as determined by co-expression of CD38 and HLA-DR) decreases during ART (4,16,22), probably reflecting to a large extent the reduction in HIV-specific CD8 response that accompanies the reduction in antigen load (42). However, CD8-mediated responses to CMV, for example, improve in the context of an expanding T cell repertoire (28), an effect which appears CD4 cell dependent (43).

The apparent marked dysregulation of the immune response that characterizes IRD suggests that T regulatory cell function may be an important factor in the development of IRD. An inhibitory effect on the ability of DC to mature from peripheral monocytes has been attributed to the presence of HIV-induced CD4^+^ CD25^+^ IL-10-secreting T regulatory cells (44). In addition regulatory T cells are present at higher frequency in the tonsillar tissue of untreated patients than in tissue from those receiving ART (45). It is possible that decreased T regulatory cell function during ART may enable mDC to polarize the acquired immune response towards type 1, thereby favouring development of IRD.

HIV infection is associated with polyclonal B cell activation and defective specific antibody responses (46) and ART is associated with a reduction in risk of serious bacterial infections (e.g. pneumococcal disease) in which immunity is antibody-dependent (47). Furthermore, ART improves the antibody response following vaccination or revaccination, although restoration is not always complete (48,49). There is very little information, however, about the effect of ART on eosinophil numbers and on either specific or total IgE responses that may be relevant to development of IRD in association with helminth infections.

## IMMUNOLOGICAL MECHANISMS OF IRD

IRD is most likely to develop in association with those infections for which immune responses are markedly suppressed by HIV and rapidly restored during ART. It occurs most frequently among patients with nadir CD4 cell counts < 50 cells/µL (8,10) who frequently have either subclinical infections or suppressed responses to clinical disease and yet also retain capacity for rapid increments in immune function (21). IRD results from exaggerated host inflammatory responses to soluble antigen, live organisms (in the case of subclinical or partially treated infections) or dead organisms (in the case of treated infections) (8,10,15). It is also recognized that antigen may persist on DC for long periods, a phenomenon recently demonstrated *in vivo* for HIV antigen despite successful ART (50). When viable organisms are present, it may be difficult to distinguish between IRD and active opportunistic infection, including that resulting from drug resistance. Whilst antigen concentration is likely to influence the risk and severity of IRD, this has not been formally studied.

The vast majority of cases of IRD develop in the first 3 months of ART (8,10), corresponding to the first phase of immune reconstitution in which there is a very rapid increase in both the number of circulating of CD45RO^+^ memory cells as well as CD4 cell function (4). The extent of sequestration of activated CD45RO^+^ memory cells in lymphoid tissue may be directly related to the HIV load, which would explain why those with the greatest pretreatment viral loads have the greatest phase 1 rates of CD4 cell recovery during ART (18,19) and the greatest risk of IRD (51,52). Recirculation of this previously sequestered cell population may provide the opportunity for relevant pathogen-specific cells to gain access to sites of infection and engage in the host inflammatory response to foreign antigen. IRD can develop within the first 1–2 weeks of ART even prior to any detectable increase in circulating CD4 cell numbers, and this is likely to reflect rapid improvements in immune function.

Most cases of IRD are associated with chronic bacterial infections, viral infections and deep fungal infections, which typically trigger immunopathology via cell-mediated T-helper type 1 (T_H_1) cytokine-secreting immune responses (8,10,53). Much of our knowledge about the mechanisms of IRD comes from study of cases of mycobacterial IRD (10). Development of IRD coincides with restoration of T lymphocyte proliferative responses, IFN-γ secretion and cell-mediated immune responses to mycobacteria, leading to restoration of delayed type hypersensitivity skin test responses to mycobacterial antigens (29,54,55). These processes are associated with expansion in the number of antigen-specific T cells during IRD (53). Reports of hypercalcaemia (resulting from autologous production of 1,25 dihydroxycholecalceriferol) as a manifestation of mycobacterium-associated and cryptococcal IRD also provide evidence of restoration of the physiological function of granulomas (56,57). As might be expected, IRD associated with viral infections, such as cytomegalovirus, JC virus, varicella-zoster virus and hepatitis C virus, is associated with restoration of CD8 T cell responses (58–61). However, data regarding mechanisms of IRD associated with other types of opportunistic infections are lacking.

What determines whether a patient develops immune responses that effectively clear infection vs. responses that lead to the development of florid immunopathology is not clear. It is perhaps the rapidity with which cell-mediated immune responses are restored and a lack of compensating immunoregulatory mechanisms may lead to the uncontrolled tissue-damaging responses that characterize IRD. In this respect, it has been speculated that some patients may have a genetically determined immunological predisposition to development of IRD (62). It is hypothesized that polymorphisms in cytokine genes may influence the rate of clearance of opportunistic pathogens or may cause dysregulation of the inflammatory response. Association of such polymorphisms with development of IRD has been described in small groups of patients developing IRD associated with a range of organisms (62). However, whether mechanistic relationships exist has yet to be demonstrated.

## IRD ASSOCIATED WITH PARASITIC INFECTIONS

The number of reports of IRD associated with parasitic diseases is small but increasing (Table 1) and most have been among immigrants to developed countries. Several reasons may underlie this paucity of data. The association of parasitic infections with IRD may be infrequent or may typically be mild or non-specific, leading to lack of recognition. Indeed, many parasitic infections such as schistosomiasis, trypanosomiasis and filariasis have complex systems of immune evasion, permitting these organisms to exist with little or no host inflammatory response (63). Furthermore, most parasitic diseases induce T-helper type 2 cytokine-secreting immune responses, mediated by antibody production and eosinophils. While such responses may be augmented by restoration of appropriate T-helper cell function during ART, the resulting effects on acute inflammatory processes are perhaps less likely to be clinically apparent compared to those resulting from augmentation of type 1 immune responses. Other reasons for the lack of reported cases of IRD associated with parasitic infections may include lack of awareness of health care personnel or lack of facilities in resource-limited settings to establish diagnoses. However, as clinical awareness of the spectrum of IRD increases and experience with ART grows in resource-limited countries, cases may be recognized more frequently.

**Table 1:** Reported cases of immune reconstitution disease associated with parasitic infections

Organism	No. of cases	Clinical manifestation	Baseline CD4 cell count/µL	Presentation or deterioration	Reference
Protozoa					
*Leishmania infantum*	2	Visceral leishmaniasis	186, 15	Presentation	Berry, 2004 (68)
*Leishmania* spp.	1	Visceral leishmaniasis	–	Presentation	Albrecht, 1998 (69)
(unspecified)	3	Visceral leishmaniasis	94, 39, 30	Presentation	Jimenez-Exposito, 1999 (70)
*Leishmania braziliensis*	1	Cutaneous + mucosal leishmaniasis	38	Presentation	Posada-Vergara, 2005 (74)
	1	Cutaneous + mucosal leishmaniasis	23	Deterioration	Posada-Vergara, 2005 (74)
*Leishmania major*	1	Uveitis	4	Presentation	Blanche, 2002 (77)
*Leishmania infantum*	1	Post kala azar dermal leishmaniasis	35	Presentation	Ridolfo, 2000 (76)
*Leishmania donovani* (presumed)	1	Post kala azar dermal leishmaniasis	150	Presentation	Gilad, 2001 (75)
*Toxoplasma gondii*	1	Cerebral toxoplasmosis	83	Presentation	Tsambiras, 2005 (86)
	1	Cerebral toxoplasmosis	–	–	Jevtovic, 2001 (85)
Helminths					
*Schistosoma mansoni*	1	Eosinophilia	170	Presentation	Fernando, 2002 (83)
	1	Eosinophilic enteritis	170	Presentation	de Silva, 2006 (84)
*Strongyloides stercoralis*	1	Fever, eosinophilia and hepatitis	32	Presentation	Kim, 2004 (81)
	1	Disseminated strongyloidiasis	135	Presentation	Lanzafame, 2005 (82)

### Leishmaniasis

Visceral disease (VL) is the clinical form of leishmaniasis that has the most substantial geographical overlap with HIV and typically occurs as an opportunistic infection among HIV-infected individuals with CD4 cell counts < 200 cells/µL (64). This overlap is predominantly in southern Europe and the horn of Africa but expansion of the HIV epidemic in India is also likely to result in an increasing number of co-infected individuals there. In both HIV-infected and non-infected patients, large numbers of parasites multiply within phagocytic mononuclear cells and induce a type 2 cytokine response and hypergammaglobulinaemia (65). T cell proliferation and IFN-γ production on stimulation with leishmania antigen *in vitro* are typically deficient (66).

Many patients with VL have received ART in southern Europe (67) and yet the number of suspected cases of IRD is very few. It is notable that all six of the possible cases describe the unmasking of subclinical infection following initiation of ART, whereas none of the reports describe clinical deterioration of pre-existing VL (Table 1). Two reports of IRD associated with *L. infantum* VL in southern France describe asymptomatic patients who developed fever and hepatosplenomegaly due to VL within the first 2 weeks of initiating ART (68). Another report describes a patient who had not been to a leishmaniasis-endemic area for 8 years and who, following commencement of ART, soon developed fever and thrombocytopenia due to VL (69). A further report describes three patients in Spain who had an excellent virological response to ART and yet developed fever and hepatosplenomegaly during the initial months of ART (70). However, an important study by de la Rosa *et al*. suggests that this phenomenon is uncommon (71). They described a series of 11 patients with documented untreated subclinical VL who commenced ART; none developed clinical VL during follow-up (71), suggesting that the risk of development of VL-associated IRD among such patients is low.

Two factors are leading to increased overlap of New World leishmaniasis and HIV infections in South America: a rising prevalence of leishmaniasis in urban areas as well as expansion of the HIV epidemic into rural areas where leishmaniasis is more common. Cutaneous, mucosal and post kala azar dermal leishmaniasis (PKDL) are associated with an intense granulomatous response, causing tissue pathology via a CD4 cell-dependent process (72,73). From a mechanistic viewpoint, one might therefore expect that IRD would be more commonly associated with tegumentary forms of disease rather than VL. However, as yet reports of the former are few.

Two cases of IRD associated with cutaneous and mucosal leishmaniasis have been reported from Brazil (Table 1) (74). One patient, who had left an area endemic for leishmaniasis 15 years earlier, developed progressive cutaneous papules, plaques and ulcers and also ulceration of the oronasopharyngeal mucosa after initiating ART. The second HIV-infected patient presented with multiple cutaneous leishmaniasis lesions, which deteriorated markedly 1 month after starting ART and which were also accompanied by development of oronasopharyngeal lesions. Diagnoses of disease due to *Leishmania braziliensis* were established in both patients and both responded well to treatment with either amphotericin B or pentavalent antimony (74).

Two cases of PKDL presenting as IRD have been reported from Israel (75) and Italy (76). An Ethiopian immigrant to Israel who had been treated for VL some years earlier developed the typical cutaneous lesions of PKDL over the face and torso 2 weeks after initiating ART (75). The diagnosis was histologically confirmed. The other case was an HIV-infected patient who was initially treated for VL and then developed PKDL on her lower limbs 6 months after commencing ART (76). However, a diagnosis of IRD in this latter case cannot be strongly justified and the presentation may simply represent the normal presentation of PKDL.

A further patient with diffuse cutaneous leishmaniasis in France treated successfully with amphotericin B developed severe bilateral granulomatous anterior uveitis 4 months after starting ART (77). Treatment with liposomal amphotericin B and interferon-γ (IFN-γ) failed to control the disease and one eye had to be enucleated. Histological examination revealed florid inflammation and the presence of amastigotes. A further similar case of fulminant ocular leishmaniasis due to *Leishmania donovani* during ART has been reported from the Netherlands (78). Histological examination of the enucleated eye revealed massive granulomatous infiltrates throughout the eye but the distinction between active infection and IRD was unclear.

### Strongyloidiasis

The lack of an association between HIV-related immunodeficiency and risk of disseminated strongyloidiasis has been intriguing (79). A possible explanation for this is suggested by data reported by Viney *et al*., who demonstrated that progressive HIV-associated CD4 lymphocytopenia was associated with diminishing likelihood of larval maturation within the gut. Decreased larval maturation would diminish the risk of autoinfection – a process required in the development of hyperinfection (80). However, two cases of possible IRD involving disseminated strongyloidiasis have now been reported, representing a new and unexpected interaction (81,82). Both were in immigrants from low-income to high-income countries and presented within the first weeks of ART. The first case developed fever, eosinophilia and hepatitis (81); the second patient presented with gastrointestinal symptoms and pruritis, and investigations revealed pulmonary radiographic infiltrates and eosinophilia (82). Numerous larvae of *Strongyloides stercoralis* were found in stool specimens from both patients and both responded well to antihelminthic treatment. Both cases were temporally related to the commencement of ART.

It is not clear whether these cases arose due to the triggering of immune responses to pre-existing disseminated infection in these patients or whether immune recovery facilitated dissemination of strongyloidiasis. Of note, the first case had recently received corticosteroids as a component of treatment for cerebral toxoplasmosis and this would have served as a potent stimulus for dissemination of the strongyloides infection prior to starting ART.

### Schistosomiasis

Two cases of schistosomiasis-associated IRD have been reported (Table 1) (83,84). The first report describes an African immigrant to the UK who was found to be HIV-infected and to have hepatosplenomegaly. The baseline eosinophil count was normal but this increased 1 month after initiating ART and peaked at 1·5 × 10^9^ cells/L after 4 months of treatment. The hepatosplenomegaly was proven by biopsy to be due to *Schistosoma mansoni* infection and the patient was treated with praziquantel, leading to a reduction in eosinophil count. No clinical manifestations accompanied the eosinophilia and so this might be better termed an immune reconstitution ‘phenomenon’ rather than ‘disease’. The second report described a South African man who emigrated to the UK and 14 years later commenced ART (84). Treatment was stopped and reinitiated on five occasions due to development of vomiting, diarrhoea and abdominal pain. Symptoms developed within the first few weeks of treatment on each occasion. Colonoscopy revealed patchy inflammation of the large bowel and mucosal biopsies showed florid eosinophilic granulomatous inflammation associated with both viable and dead eggs of *S. mansoni.* No comment was made on whether the episodes were associated with peripheral blood eosinophilia. Subsequent treatment for schistosomiasis led to resolution of symptoms.

Disease associated with schistosomiasis is mediated by eosinophilic granulomatous inflammation associated with ova. As with the cases of strongyloides-associated IRD, enhancement of T helper type 2 responses may have exacerbated eosinophilic inflammation in both of these cases. If schistosoma eggs antigens were responsible for triggering the immune-mediated inflammation within the intestinal wall in the case described by de Silva *et al*. (84), it is intriguing that symptoms were resolved following praziquantel treatment, which would not have affected the existing egg antigen burden within the intestinal mucosa.

Large numbers of patients have received ART in east Africa where the prevalence of schistosomiasis is high and yet no reports of schistosomiasis-associated IRD have emerged from the region. Awareness of this potential complication of ART may lead to recognition of further cases.

### Other parasites

Just two cases of suspected IRD associated with toxoplasma encephalitis have been reported in published literature. In one case no clinical details were given (85). In the other, an HIV-infected patient with a nadir CD4 cell count of 83 cells/µL presented with a focal seizure after 3 weeks of ART (86). This was diagnosed as cerebral toxoplasmosis on the basis of positive serology, multiple ring-enhancing intracerebral lesions on a magnetic resonance imaging scan and a positive response to treatment for toxoplasmosis. Toxoplasmosis is a common end-stage opportunistic infection in industrialized countries for which ART is often initiated fairly early. The fact that more cases have not been reported casts some doubt on whether this infection may be associated with IRD.

There are no reports in the literature of IRD associated with helminthic or protozoal infections other than those described above. However, an important question is whether malaria may be associated with IRD. Although large numbers of individuals have received ART in sub-Saharan African countries where *Plasmodium falciparum* malaria is hyperendemic, no cases of malaria-associated IRD have been reported. The likeliest manifestation, however, might be very non-specific such as development of fever; it seems unlikely that severe manifestations would arise.

## CONCLUSIONS

IRD associated with infections has emerged as an important cause of morbidity complicating the initial months of ART. As access to ART is scaled up in many low-income countries, the extent to which IRD will contribute to morbidity and mortality in treatment programmes has yet to be determined. However, as patients typically present to ART services with marked lymphocytopenia and frequent co-infections, IRD is likely to be common. Most IRD results from dysregulated augmentation of cell-mediated immune responses to mycobacterial, chronic viral and invasive fungal infections. However, the spectrum of infections recognized to be associated with IRD now encompasses a number of parasitic diseases, including leishmaniasis (visceral, cutaneous, mucosal and PKDL), schistosomiasis and strongyloidiasis. No studies have yet examined the mechanisms of IRD associated with helminth infections, but these may be due to augmentation of appropriate type 2 immune responses. More research is needed to increase our understanding of these emerging phenomena.
